# *Echinococcus granulosus* Protoscoleces-Derived Exosome-like Vesicles and Egr-miR-277a-3p Promote Dendritic Cell Maturation and Differentiation

**DOI:** 10.3390/cells11203220

**Published:** 2022-10-14

**Authors:** Xiaofan Zhang, Wenci Gong, Chaohui Duan, Huixia Cai, Yujuan Shen, Jianping Cao

**Affiliations:** 1 National Institute of Parasitic Diseases, Chinese Center for Disease Control and Prevention, (Chinese Center for Tropical Diseases Research), Key Laboratory of Parasite and Vector Biology, National Health Commission of the People’s Republic of China, World Health Organization Collaborating Centre for Tropical Diseases, Shanghai 200025, China; 2 Department of Laboratory Medicine, Sun Yat-Sen Memorial Hospital, Sun Yat-Sen University, Guangzhou 510120, China; 3 Department of Parasite Control, Qinghai Province Institute for Endemic Diseases Prevention and Control, Xining 811602, China; 4 The School of Global Health, Chinese Center for Tropical Diseases Research, Shanghai Jiao Tong University School of Medicine, Shanghai 200025, China

**Keywords:** *Echinococcus granulosus*, exosome-like vesicles, microRNA, egr-miR-277a-3p, dendritic cells

## Abstract

Cystic echinococcosis, a major parasitic disease caused by *Echinococcus granulosus*, seriously threatens human health. The excretory–secretory (ES) products of *E. granulosus* can induce immune tolerance in dendritic cells (DCs) to downregulate the host’s immune response; however, the effect of exosomes in the ES products on the DCs has remained unclear. This study showed that *E. granulosus* protoscoleces-derived exosome-like vesicles (PSC-ELVs) could be internalized by bone marrow-derived dendritic cells (BMDCs), allowing for the delivery of the parasite microRNAs to the BMDCs. Moreover, PSC-ELVs induced BMDCs to produce the proinflammatory cytokinesinterleukin (IL)-6, IL-12, IL-β, tumor necrosis factor-alpha (TNF-α), and interferon-gamma (IFN-γ). PSC-ELVs also upregulated the BMDCs surface marker major histocompatibility complex class II (MHC II), as well as costimulatory molecules CD40, CD80, and CD86. PSC-ELV-derived egr-miR-277a-3p upregulated the *IL-6*, *IL-12*, and *TNF-α* mRNA levels in BMDCs. Moreover, egr-miR-277a-3p directly targeted *Nfkb1* (encoding nuclear factor kappa B 1) to significantly suppress the mRNA and protein levels of NF-κB1 in BMDCs, while the expression of NF-κB p65 significantly increased, suggesting that egr-miR-277a-3p induces the production of proinflammatory cytokines by the modification of the NF-kB p65/p50 ratio in BMDCs. These results demonstrated that PSC-ELVs and egr-miR-277a-3p might enhance DCs maturation and differentiation in a cross-species manner, which in turn may modulate the host immune responses and offer a new approach to echinococcosis prevention and treatment.

## 1. Introduction

Cystic echinococcosis is a serious parasitic disease caused by *Echinococcus granulosus* larvae, which mainly parasitize the liver and lungs of animals and humans [1]. *E. granulosus* has evolved complex strategies to escape host immune responses, primarily by directing and manipulating the immune response towards tolerance or anergy. Recent research has demonstrated numerous immunoregulatory mechanisms related to macrophages, dendritic cells (DCs), and regulatory T cells (Tregs) [2,3,4]. The early immune response of *E. granulosus* infection shows Th1-oriented Th2-type responses [5]. *E. granulosus* oncospheres and cysts could modulate DCs maturation and alter monocyte differentiation to escape the host’s immunosurveillance and promote chronic infection [6]. Prior research by the study investigators showed that the numbers of macrophages, DCs, Tregs, and myeloid-derived suppressor cells (MDSCs) increased in BALB/c mice infected by *E. granulosus* [2,4], and the expression of arginase-1 (ARG-1) in many kinds of myeloid cells was enhanced, which inhibited the T cell response to *E. granulosus* antigens [4]. It was also previously found that the *E. granulosus* excretory–secretory (ES) products could induce immune tolerance in DCs to impair the host’s immune response [7]; however, whether some components of the ES products such as microRNAs (miRNAs) could promote the differentiation and maturation of DCs remains unclear.

Excretory–secretory products play a vital role in parasite–host interactions and can downregulate the host’s immune response to mediate immunosuppression and help parasites establish infection [8]. Exosomes are a key component of ES products, comprising a type of membranous vesicles approximately 30–150 nm in diameter, which are released into the extracellular matrix following the fusion of intracellular multivesicular bodies with the cell membrane [9]. Parasite-derived exosomes are involved in the exchange of information among parasites, as well as in the interaction between parasites and their hosts. Parasite-derived exosomes can transmit differentiation, virulence, and drug-resistance genes among parasites [10]. They can also regulate the host’s gene expression and immune responses, in addition to participating in parasite pathogenesis [10]. Exosomes can be effectively internalized by the multiple cells of host, and the biologically active substances they carry such as proteins, lipids, and nucleic acids can then act on those cells, thereby regulating immune responses. Exosomes are rich in noncoding RNAs, especially miRNAs, which can specifically bind to target mRNAs, thus causing mRNA degradation or the inhibition of protein translation and resulting in the downregulation of target protein production. Strikingly, parasite and even plant miRNAs have been detected in animal bodies and human fluids and observed to regulate host genes in a cross-species manner [11]. For example, *Schistosoma* miR-2162 and miR-1 promote host liver fibrosis by directly targeting the host transforming growth factor beta (TGF-β) receptor III and frizzled-related protein 1, respectively [12,13]. Parasite-derived exosomes can carry a large amount of parasite miRNA, which is involved in the interaction between the parasite and the host, and play a vital role in parasite invasion and infection, pathogenesis, and immune evasion.

Primary immune responses are initiated by DCs, which can powerfully activate naive T cells and determine the direction of the body’s immune responses. The process of parasitic infection can induce the production of Tregs and regulatory DCs and inhibit the proliferation and differentiation of T cells [14]. Exosomes from different sources can carry a variety of biological substances, which DCs can internalize to mediate the immune response. Tumor-derived exosomes can influence the differentiation, maturation, and functions of DCs. For example, colon cancer-derived exosomal miR-155 promotes the differentiation and maturation of DCs, which then promote lymphocyte proliferation and induce Th1-type immune responses [15]. Moreover, miR-142 and Let-7i-modified breast cancer exosomes also promote DCs differentiation and maturation [16]. Both *E. granulosus* protoscoleces (PSCs) and hydatid cysts can release exosome-like vesicles [17,18]. However, the role and mechanism of *E. granulosus* exosome-derived miRNAs in the differentiation and maturation of DCs remain unclear.

Herein, the proportion of DCs in the liver leukocytes of *E. granulosus*-infected mice was found to be significantly increased at 6-, 9-, and 12-months post-infection. *E. granulosus* PSC-derived exosomes-like vesicles (PSC-ELVs) could be internalized by mouse bone marrow-derived dendritic cells (BMDCs), allowing the cargo parasite miRNAs to be transported into the BMDCs. Further studies showed that PSC-ELVs promoted the differentiation and maturation of BMDCs, which might help to induce a Th1-type immune response. Moreover, PSC-ELV-derived egr-miR-277a-3p could upregulate the mRNA levels of proinflammatory cytokines (interleukin (*IL*)-6, *IL-12*, and tumor necrosis factor-alpha (*TNF-α)*) in BMDCs. In addition, egr-miR-277a-3p significantly suppressed the mRNA and protein levels of nuclear factor kappa B1 (NF-κB1) in BMDCs by directly targeting the 3′ untranslated region (UTR) of *Nfkb1*, while the mRNA and protein levels of NF-κB P65 were significantly increased, which suggests that egr-miR-277a-3p might induce the production of proinflammatory cytokines by the modification of the NF-kB p65/p50 ratio in BMDCs.

## 2. Materials and Methods

### 2.1. Mice and Parasite Infections

Female BALB/c mice (6–8 weeks old) were bought from the SLAC Laboratory (Shanghai, China). *E. granulosus* PSCs were collected from fertile hydatid cysts of naturally infected sheep in the Xinjiang Uygur Autonomous Region, China. PSCs were washed and the viability determined according to the methods of our previous study [19]. BALB/c mice (n = 30) in the infection group were intraperitoneally inoculated with 2000 live PSCs within 200 μL of sterile 0.9% sodium chloride (NaCl), and the control group mice were inoculated with same volume of NaCl. All mice received standard laboratory food and water and were raised in specific pathogen-free conditions.

### 2.2. In Vitro Culture of Protoscoleces

PSCs (n = 20,000) were cultured in Roswell Park Memorial Institute (RPMI) 1640 medium (Gibco/Life Technologies, Grand Island, NY, USA) supplemented with glucose (4 mg/mL) and antibiotics (200 μg/mL of streptomycin and 200 U/mL of penicillin (Invitrogen, Waltham, MA, USA)) in 75-cm^2^ cell culture flasks in an incubator (37 °C, 5% CO_2_). The trypan blue dye exclusion assay was applied to detect the viability of PSCs. We harvested and changed the culture medium of PSCs every 12 h. A total of 300 mL of culture supernatant was collected when PSCs were cultured in serum-free medium for 7 days and then was stored at −80 °C until use.

### 2.3. Exosome-like Vesicle Purification and Characterization

PSC-ELVs were isolated from the PSCs culture supernatant and enriched with differential centrifugation as our previous study described [19]. Transmission electron microscopy (TEM, HITACHI, Tokyo, Japan) and NanoSight LM10 (NanoSight, Malvern Panalytical Ltd., Malvern, UK) analyses were used to detect the morphology and size distribution of the PSC-ELVs, as previously described [19].

### 2.4. Determination of the Total Protein Concentration of PSC-ELVs

The total protein concentration of the PSC-ELVs was detected with a bicinchoninic acid (BCA) protein measurement kit (Takara, Shiga, Japan). Before measurement, reagents A and B of BCA were mixed thoroughly at a ratio of 100:1 to prepare the working solution. The bovine serum albumin (BSA) standard solution was diluted with deionized water to 0, 125, 250, 500, 750, 1000, 1500, and 2000 μg/mL. Next, 25 μL of each diluted BSA standard solution and PSC-ELVs was added to a 96-well plate and 200 μL of the working solution was then added to each well and mixed immediately. The plate was put in the incubator at 37 °C for 30 min and then cooled to room temperature. The optical density of each concentration of BSA standard solution and PSC-ELVs was detected at 562 nm with a Microplate Reader (Thermo Fisher Scientific, Waltham, MA, USA). The standard curve of the BSA standard solution was drawn by subtracting the average absorbance value of the blank well (Figure S1) and then using that value to calculate the protein concentration of the PSC-ELVs.

### 2.5. Preparation and Treatment of Bone Marrow-Derived Dendritic Cells (BMDCs)

BMDCs were prepared according to the methods of a previous study, with minor modifications [20]. Briefly, bone marrow cells were isolated from the leg bones of wild-type BALB/c mice and then cultured in RPMI 1640 supplemented with 10% fetal bovine serum (Gibco), 2 ng/mL of IL-4 (PeproTech, Cranbury, NJ, USA) and 20 ng/mL of granulocyte-macrophage colony-stimulating factor (PeproTech) and maintained in an incubator (37 °C, 5% CO_2_). The medium was renewed after the initial bone marrow cells were cultured for 4 days. The cells were collected on the 7th day, and the purity of the CD11b^+^CD11c^+^ cells (BMDCs) was >95%, as detected with flow cytometry. BMDCs were treated as immature DCs in the experiments. For stimulation, 2.5 μg/mL of PSC-ELVs was added to the BMDCs after culture for 7 days, and the cells were then collected at different times according to the experimental requirements. BMDCs were transfected with 20 μΜ of egr-miR-277a-3p mimic (RiboBio, Guangzhou, China), 20 μΜ of egr-miR-277a-3p inhibitor (RiboBio), or negative controls (NC, RiboBio), according to the manufacturer’s protocols.

### 2.6. Uptake of PSC-ELVs by BMDCs

To investigate whether the PSC-ELVs could be internalized by the host BMDCs, PSC-ELVs were stained with the fluorescent lipid dye PKH67 (Sigma-Aldrich, St. Louis, MO, USA) according to the methods of a previous study with minor modifications [21]. Briefly, the PKH67-labeled PSC-ELVs (5 μg) were washed with phosphate-buffered saline (PBS) and concentrated by ultracentrifugation at 110,000× *g* for 90 min to remove the non-absorbed lipid dye. BMDCs were seeded in a 6-well plate and then co-incubated with the labeled PSC-ELVs (2.5 μg/mL) for 10 min. Meanwhile, an equal volume of PBS was co-incubated with the BMDCs as a control group. Following a 10min incubation, the culture medium was removed and BMDCs washed twice with PBS. Then, 4% paraformaldehyde was added to fix the cells for 20 min. The cell nuclei were stained with 4′6-diamidino-2-phenylindole (DAPI, Beyotime Biotechnology, Jiangsu, China). Finally, the internalization of the PSC-ELVs by the host BMDCs was observed under a confocal fluorescence microscope (Nikon, Tokyo, Japan).

### 2.7. Quantitative Real-Time Reverse Transcription-PCR (qRT-PCR)

The total small RNA in PSC-ELVs was extracted with an miRNeasy Serum/Plasma Kit (Qiagen, Hilden, Germany) and immediately reversed transcribed into cDNA with a miScript II RT Kit (Qiagen). The levels of parasite miRNA were detected using quantitative real-time PCR (qPCR) employing a QuantiFast SYBR Green PCR Kit (Qiagen). The primers used for the qPCR step of the qRT-PCR protocol are listed in Supplemental Table S1. U6 snRNA was used as an endogenous control to normalize the qPCR data, and the relative levels of miRNA were analyzed with the 2^–ΔΔCt^ method [22].

The total RNA in BMDCs was extracted with TRIzol reagent (Invitrogen) and then reversed transcribed into cDNA using a PrimeScript™ RT reagent Kit with gDNA Eraser (Takara). The cDNA was used as a template in a qPCR reaction system containing TB Green Premix Ex Taq (Takara) with 0.4 μΜ of forward and reverse primers. The primers used for the qPCR detection of mRNA are listed in Supplemental Table S2. The relative gene expression values were normalized to that of *Gapdh* (encoding glyceraldehyde-3-phosphate dehydrogenase) and analyzed with the 2^–ΔΔCt^ method.

### 2.8. Western Blotting Analysis

The treated BMDCs were washed twice with precooled PBS and then lysed with a Radioimmunoprecipitation (RIPA) lysis solution containing phosphatase and protease inhibitors on ice for 30 min. The cell lysates were separated using 10% sodium dodecyl sulfate-polyacrylamide gel electrophoresis (SDS-PAGE) and then transferred to a polyvinylidene difluoride (PVDF) membrane (Merck Millipore, Darmstadt, Germany). After blocking nonspecific binding sites, the membranes were incubated with different primary antibodies [anti-14-3-3 zeta/delta, anti-enolase-1, anti-CD9, anti-NF-κB1, anti-NF-κB p65, and anti-GAPDH (Cell Signaling Technology, MA, USA)] and their respective horseradish peroxidase (HRP)-conjugated secondary antibodies. The membranes were visualized by an chemiluminescence (ECL) detection system (Merck Millipore). The intensities of the immunoreactive protein bands were analyzed with Image J (NIH, Bethesda, MD, USA).

### 2.9. Enzyme-Linked Immunosorbent Assay (ELISA)

BMDCs were stimulated with PSC-ELVs for 12 h. Then, the protein levels of IL-6, IL-12p70, IL-1β, TNF-α, interferon-gamma (IFN-γ), and IL-10 were detected in the supernatants of BMDCs using ELISAs (eBioscience, San Diego, CA, USA) following the manufacturer’s instructions.

### 2.10. Flow Cytometry

Flow cytometry was used to detect the expression of major histocompatibility complex class II (MHC II) and costimulatory molecules on the surface of BMDCs stimulated by PSC-ELVs. The treated BMDCs were incubated with the following fluorescently labeled antibodies at 4 °C for 30 min: BV421-labeled anti-CD11b, FITC-labeled anti-CD11c, PE-labeled anti-MHC II, APC-labeled anti-CD86, PE-Cy7-labeled anti-CD80, and PE-Cy7-labeled anti-CD40 (BioLegend, San Diego, CA, USA). An LSRFortessa X-20 instrument (BD Biosciences, Franklin Lakes, NJ, USA) and FlowJo software (Tree Star Inc., Ashland, OR, USA) were used to acquire and analyze the flow cytometry data.

### 2.11. 3′ UTR Luciferase Reporter Constructs

According to the online miRDB database, the seed sequence of egr-miR-277a-3p was predicted to be complementary with the 3′ UTR of *Nfkb1*. Wild-type and mutant 3′ UTRs of *Nfkb1* containing the predicted egr-miR-277a-3p binding sites were chemically synthesized and then cloned into the pmirGLO vector (Promega, Madison, WI, USA). Seeded in a 96-well plate, the 293T cells were transfected with 50 nM of egr-miR-277a-3p mimics or a negative control (RiboBio) and were co-transfected with 250 ng of wild-type or mutant *Nfkb1* 3ʹ UTR plasmid, using Lipofectamine 2000 (Invitrogen) following the manufacturer’s protocols. The cells were collected after transfection for 48 h, and the luciferase activity of the *Nfkb1* 3ʹ UTR plasmid was detected with a Dual-Glo Luciferase Assay System (Promega).

### 2.12. Statistical Analysis

Data are presented as the mean ± SD and were analyzed with an unpaired Student’s *t*-test or one-way analysis of variance (ANOVA) using GraphPad Prism version 7.0 (GraphPad Software Inc., San Diego, CA, USA). Differences were considered statistically significant when the *p*-value was less than 0.05.

## 3. Results

### 3.1. Identification of E. granulosus Protoscoleces-Derived Exosome-like Vesicles (PSC-ELVs)

**Figure 1:**
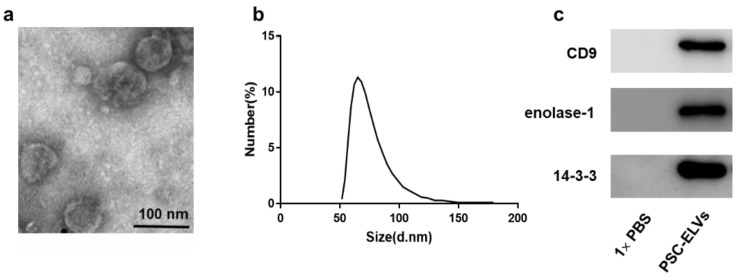
Identification of *E. granulosus* protoscoleces-derived exosome-like vesicles (PSC-ELVs). (**a**) Images of the rounded or cup-shaped PSC-ELVs under transmission electron microscopy (TEM). Scale bars: 100 nm; (**b**) The size distribution of the PSC-ELVs. (**c**) Western blotting analysis of CD9, enolase-1, and 14-3-3 in PSC-ELVs.

Transmission electron microscopy (TEM) and nanoparticle tracking analysis (NTA) were performed to evaluate the morphology and size distribution of the PSC-ELVs. Rounded or cup-shaped vesicles with a diameter of 30–150 nm were observed under TEM (Figure 1a). NTA also demonstrated that the most purified vesicles isolated from PSCs were between 60–80 nm in diameter (Figure 1b), which was consistent with the particle size of exosomes [23]. Western blotting analysis further confirmed that the exosomal marker proteins, including CD9, enolase-1, and 14-3-3 zeta/delta, were present in the PSC-ELVs (Figure 1c).

### 3.2. Internalization of E. granulosus miRNAs in Host BMDCs via PSC-ELVs

**Figure 2:**
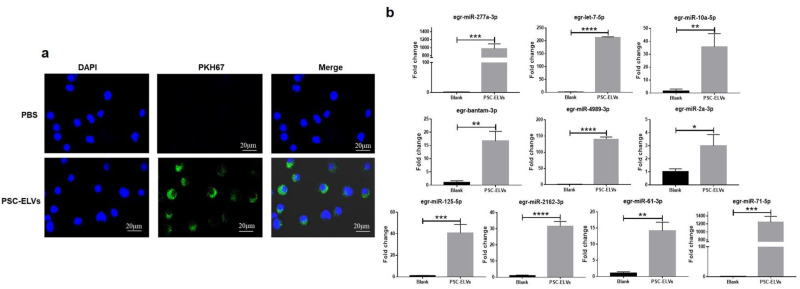
PSC-ELVs were internalized by mouse bone marrow-derived dendritic cells (BMDCs), and parasite miRNAs were transferred into the BMDCs. (**a**) BMDCs and PSC-ELVs were stained with a nuclear marker (DAPI, blue) and PKH67 (green) and were viewed under a confocal microscope (magnification 40×) Scale bars: 20 μm; (**b**) Quantitative real-time reverse transcription-PCR (qRT-PCR) analysis of parasite miRNAs in BMDCs after PSC-ELVs co-culture. * *p* < 0.05; ** *p* < 0.01; *** *p* < 0.001; **** *p* < 0.0001.

Exosomes play a vital role in host–parasite interactions. As such, the current study investigators previously isolated PSC-ELVs and identified the ncRNA profiles that might be involved in the regulation of host immunity [19]. Egr-miR-277a-3p was found to be one of the most abundant parasite miRNAs in PSC-ELVs. To confirm whether *E. granulosus* PSC-ELVs can be internalized by host cells, the PSC-ELVs were labeled with the lipid dye PKH67, and the labeled exosomes were incubated with BMDCs in vitro. The results showed that the PSC-ELVs could be internalized by BMDCs (Figure 2a). To further observe whether PSC-ELVs can transport the *E. granulosus* miRNA cargo into BMDCs, qRT-PCR was performed to detect the levels of the 10 most abundant miRNAs found in PSC-ELVs in BMDCs. After being incubated with PSC-ELVs for 4 h, the levels of the 10 most abundant miRNAs (egr-miR-277a-3p, egr-bantam-3p, egr-let-7-5p, egr-miR-4989-3p, egr-miR-10a-5p, egr-miR-2162-3p, egr-miR-125-5p, egr-miR-71-5p, egr-miR-61-3p, and egr-miR-2a-3p) in BMDCs were found to be significantly increased (Figure 2b). These data suggested that PSC-ELVs can be effectively internalized by BMDCs, meanwhile, *E. granulosus* miRNAs such as egr-miR-277a-3p can be transferred into BMDCs.

### 3.3. PSC-ELVs Promote the Differentiation and Maturation of BMDCs

**Figure 3:**
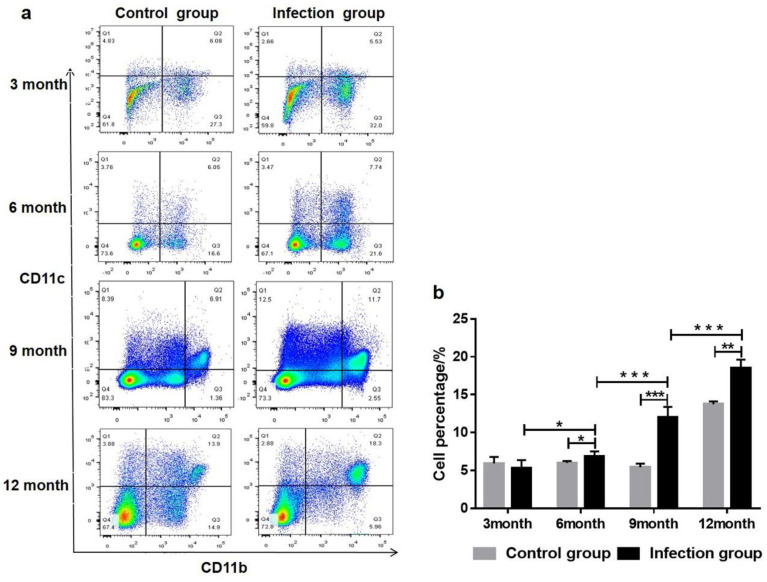
The percentage of dendritic cells (DCs) in the liver leukocytes of mice at each stage post-*E. granulosus* infection. (**a**) Flow cytometry detection of the percentage of DCs in the liver leukocytes of mice at 3, 6, 9, and 12 months post-infection; (**b**) Statistical analysis of the percentage of DCs. * *p* < 0.05, ** *p* < 0.01, *** *p* < 0.001.

DCs are powerful antigen-presenting cells that can effectively initiate the body’s primary immune response and further determine the direction of the body’s immune response during a parasitic infection. To profile the dynamic changes of DCs, flow cytometry was performed to detect the ratio of DCs in the liver leukocytes of mice after infection with *E. granulosus* (at 3, 6, 9, and 12 months). Compared with the control group, the level of DCs in liver leukocytes was increased at 6, 9, and 12 months post-infection (F_(3, 22)_ = 201.4, *p* < 0.0001) and exhibited an upward trend for the duration of the infection (Figure 3a,b). To further study the role of *E. granulosus* PSC-ELVs in the differentiation and maturation of BMDCs, the expression levels of cytokines and surface molecules of BMDCs were detected after incubation with PSC-ELVs.

**Figure 4:**
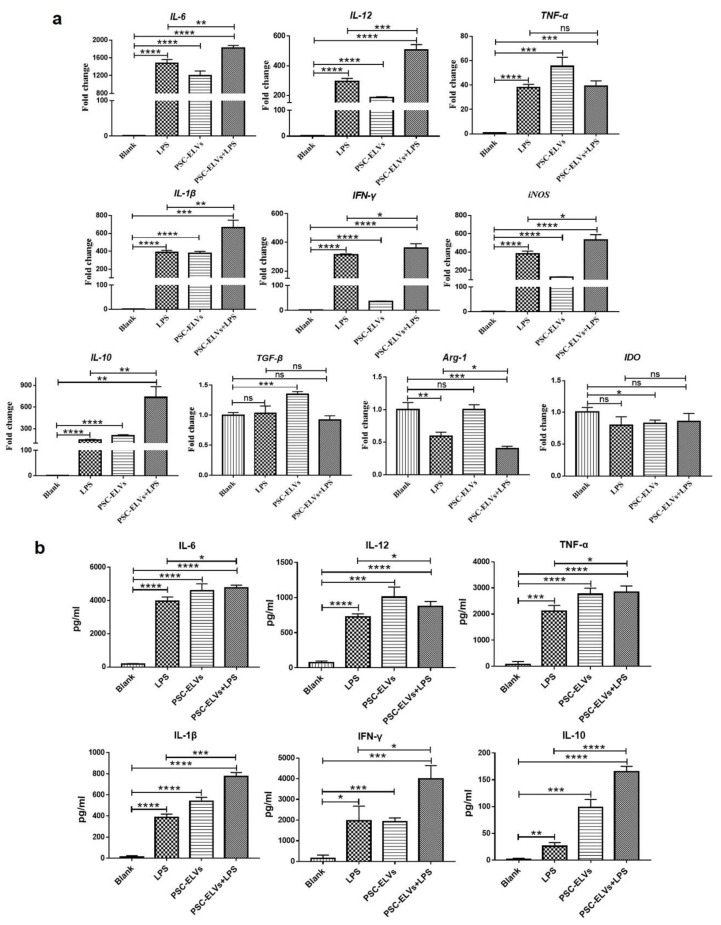
The expression levels of proinflammatory and anti-inflammatory factors in BMDCs stimulated by PSC-ELVs. (**a**) The mRNA levels of proinflammatory and anti-inflammatory factors in BMDCs stimulated with PSC-ELVs, as detected using qRT-PCR; (**b**) The protein levels of proinflammatory and anti-inflammatory factors in BMDCs stimulated with PSC-ELVs, as detected using enzyme-linked immunosorbent assay (ELISA). * *p* < 0.05; ** *p* < 0.01; *** *p* < 0.001; **** *p* < 0.0001; ns, not significant.

BMDCs were stimulated with PSC-ELVs (2.5 μg/mL) and lipopolysaccharide (LPS, 100 ng/mL). After 4 h, the BMDCs were collected to extract total cellular RNA, and then qRT-PCR was performed to determinate the mRNA levels of cytokines in the BMDCs. The results showed that compared with the blank control group, PSC-ELVs could effectively upregulate the mRNA levels of proinflammatory factors *IL-6*, *IL-12*, *TNF-α*, *IL-1β*, and *IFN-γ* and inducible nitric oxide synthase (*iNOS*), as well as anti-inflammatory factors *IL-10* and transforming growth factor beta (*TGF-β*) in BMDCs, while they downregulated the mRNA levels of anti-inflammatory factor indoleamine 2,3-dioxygenase 1 (*IDO*) (Figure 4a). In general, the mRNA levels of proinflammatory factors increased significantly and showed clear proinflammatory characteristics. Compared with the LPS-only group, co-stimulation with PSC-ELVs and LPS had a synergistic effect, inducing BMDCs to increase the mRNA levels of *IL-6*, *IL-12*, *IL-1β*, *iNOS*, *IFN-γ*, and *IL-10* (Figure 4a). Moreover, ELISA was used to further determine the secretion of cytokines in the supernatant of BMDCs after incubation with PSC-ELVs or LPS for 24 hours. The ELISA results suggested that compared with the blank control group, PSC-ELVs could effectively induce BMDCs to produce high levels of the proinflammatory factors IL-6, IL-12, TNF-α, IL-1β, and IFN-γ, as well as low levels of the anti-inflammatory factor IL-10 (Figure 4b). Compared with the LPS-only group, co-stimulation by PSC-ELVs and LPS induced higher levels of IL-6, IL-12, TNF-α, IL-1β, IFN- γ, and IL-10, thus exhibiting a synergistic effect (Figure 4b). This result was consistent with the results of qRT-PCR. Overall, PSC-ELVs caused BMDCs to produce an inflammatory immune response, inducing a stimulatory DCs polarized phenotype in vitro and demonstrating a synergistic effect with LPS.

**Figure 5:**
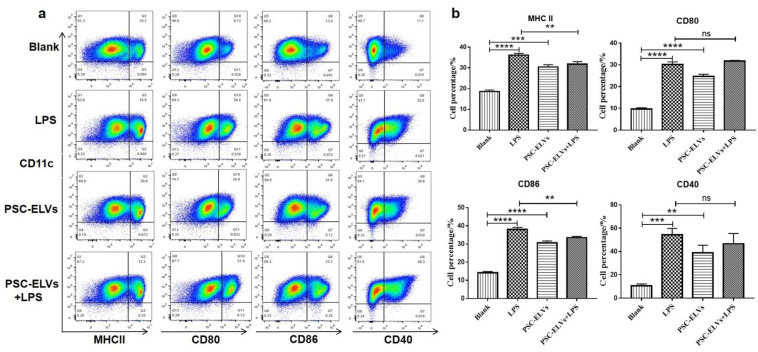
The changes in the expressions of major histocompatibility complex class II (MHC Ⅱ), CD40, CD80, and CD86 on the surface of BMDCs stimulated with PSC-ELVs, as detected using flow cytometry. (**a**) Pseudocolor diagrams of the BMDCs surface markers; (**b**) Statistical analysis of the proportions of BMDCs surface molecules, ** *p* < 0.01; *** *p* < 0.001; **** *p* < 0.0001; ns, not significant.

DCs are a type of professional antigen-presenting cell, and their surface MHC Ⅱ and costimulatory molecules play a vital role in the process of antigen presentation. To explore whether PSC-ELVs affect the expression levels of BMDCs surface MHC II and costimulatory molecules, flow cytometry was applied to detect the levels of BMDCs surface markers after stimulation with 2.5 μg/mL PSC-ELVs or 100 ng/mL LPS for 24 h. PSC-ELVs were found to effectively upregulate the expression of MHC II molecules as well as that of CD40, CD80, and CD86 on BMDCs (Figure 5a,b). Compared with the LPS-only group, co-stimulation by PSC-ELVs and LPS downregulated the expression of MHC II and CD86 but caused no significant changes in the other co-stimulatory molecules (Figure 5). Thus, these results suggested that PSC-ELVs promote the differentiation and maturation of BMDCs.

### 3.4. Egr-miR-277a-3p Induces BMDCs to Upregulate the Expression of Inflammatory Cytokines

**Figure 6:**
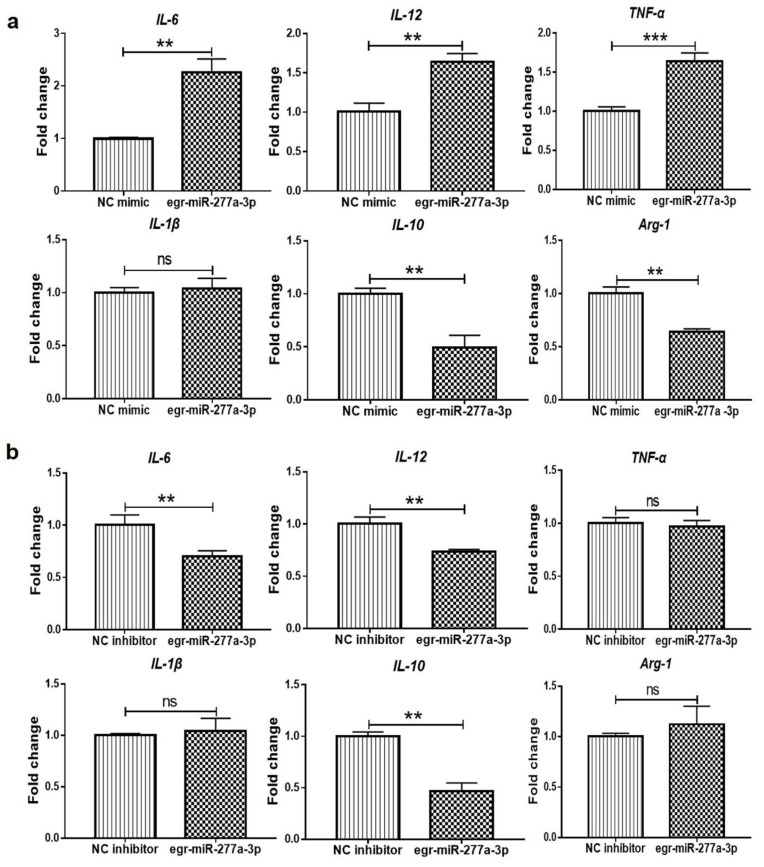
The mRNA levels of interleukin (*IL*)*-6*, *IL-12*, tumor necrosis factor-alpha (*TNF-a*)*, IL1-β*, *IL-10,* and arginase-1 (*Arg-1*) in BMDCs transfected with the egr-miR-277a-3p mimic and inhibitor, as detected by qRT-PCR. (**a**) The qRT-PCR results in BMDCs transfected with the egr-miR-277a-3p mimic; (**b**) The qRT-PCR results in BMDCs transfected with the egr-miR-277a-3p inhibitor. ** *p* < 0.01; *** *p* < 0.001; ns, not significant.

Egr-miR-277a-3p is one of the most abundant miRNAs in PSC-ELVs and can be transferred into BMDCs via PSC-ELVs. To study the role of egr-miR-277a-3p in PSC-ELV-mediated induction of proinflammatory cytokine production in BMDCs, an egr-miR-277a-3p mimic or inhibitor was transfected into BMDCs, and the cytokines mRNA levels were detected using qRT-PCR. Egr-miR-277a-3p is a parasite-derived miRNA; therefore, PSC-ELVs were added to provide a target for the egr-miR-277a-3p inhibitor. The results showed that the egr-miR-277a-3p mimic could upregulate the mRNA levels of *IL-6, IL-12*, and *TNF-α* in BMDCs. Meanwhile, the mRNA levels of anti-inflammatory factors *IL-10* and *ARG-1* were significantly decreased, while *IL-1β* mRNA levels showed no significant changes (Figure 6a), which showed clear proinflammatory properties overall. By contrast, in BMDCs, the egr-miR-277a-3p inhibitor induced decreased mRNA levels of *IL-6* and *IL-12*, while there was no significant change in the *IL-1β* mRNA (Figure 6b). This was consistent with the transfection results of the egr-miR-277a-3p mimic, which showed proinflammatory properties. No significant change was seen in the mRNA levels of *TNF-α* and *ARG-1* in BMDCs transfected with the egr-miR-277a-3p inhibitor. However, the egr-miR-277a-3p inhibitor downregulated the mRNA level of *IL-10* in BMDCs, which contrasted with the transfection results of the egr-miR-277a-3p mimic. These data proved that egr-miR-277a-3p could induce BMDCs to upregulate the mRNA levels of inflammatory cytokines, which might influence the differentiation and maturation of BMDCs.

### 3.5. Egr-miR-277a-3p Directly Targets Nfkb1 and Modifies the Nuclear NF-κB p65/p50 Ratio

**Figure 7:**
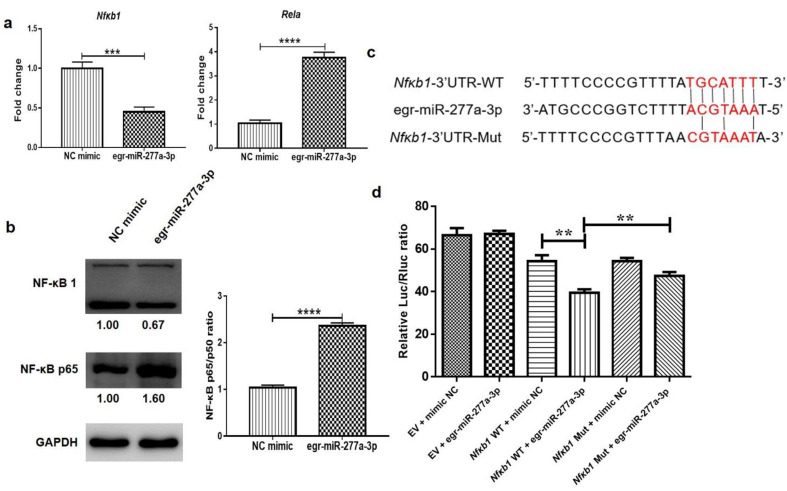
egr-miR-277a-3p directly targets *Nfkb1* (encoding nuclear factor kappa B1) and increases the NF-kB p65/p50 ratio in BMDCs. (**a**) The mRNA level of *Nfkb1* and *Rela* (encoding nuclear factor kappa B P65) in BMDCs transfected with the egr-miR-277a-3p mimic. (**b**) The protein levels of NF-κB1 and NF-κB p65 and the NF-κB p65/p50 ratio in BMDCs transfected with the egr-miR-277a-3p mimic. (**c**) Sequence alignment of egr-miR-277a-3p and its target sites in the 3ʹ untranslated region (UTR) of *Nfkb1*. (**d**) Luciferase reporter assays of 293T cells transfected with pmirGLO vectors carrying the wild-type (WT) or mutant (Mut) 3ʹ UTR of *Nfkb1* in the absence or presence of the egr-miR-277a-3p mimic (n = 3). ** *p* < 0.01; *** *p* < 0.001; **** *p* < 0.0001.

The target genes of egr-miR-277a-3p were predicted using the online bioinformatics software miRDB. Among them, *Nfkb1* was identified as a potential target gene of egr-miR-277a-3p. *Nfkb1* encodes the NF-κB p105 protein, which is processed to generate the p50 subunit of NF-ĸB. qRT-PCR and western blotting were applied to determine the mRNA and protein levels of NF-κB1 in BMDCs after transfection with the egr-miR-277a-3p mimic. qRT-PCR results suggested that the egr-miR-277a-3p mimic could suppress the mRNA levels of *Nfkb1* (Figure 7a). At the same time, the mRNA levels of *Rela* (encoding the p65 subunit of NF-κB) were significantly increased (Figure 7a). In its canonical signaling pathway, NF-κB is a heterodimer consisting of p65 and p50 subunits. The p65 protein functions as the activator of the p65-p50 heterodimer, and an increase in the NF-κB p65/p50 ratio is beneficial to the secretion of inflammatory factors TNF-α and IL-6 [24,25]. Western blotting showed that the NF-κB P65 protein levels were significantly increased after the transfection of the egr-miR-277a-3p mimic into BMDCs, while the NF-κB1 levels were significantly decreased (Figure 7b). Thus, egr-miR-277a-3p markedly increased the NF-κB p65/p50 ratio in BMDCs.

To confirm whether *Nfkb1* is a direct target of egr-miR-277a-3p, luciferase reporter constructs were generated containing wild-type or mutated *Nfkb1* 3ʹ UTR (*Nfkb1*-3ʹ UTR-WT or *Nfkb1*-3ʹ UTR-Mut) complementary sites of egr-miR-277a-3p (Figure 7c). The 293T cells were co-transfected with the constructs and either egr-miR-277a-3p mimics or the mimic NC. Luciferase activity was found to be significantly decreased in the cells transfected with the *Nfkb1*-3ʹ UTR-WT and egr-miR-277a-3p mimics compared to the cells transfected with the NC mimic (Figure 7d). Conversely, the mutation of four nucleotides in the seed-binding sequence of the *Nfkb1* 3ʹ UTR induced a complete abrogation of that inhibition and thus increased luciferase activity (Figure 7d). These data indicated that egr-miR-277a-3p directly targets *Nfkb1*, which might upregulate the expression of p65 to promote the expression of the inflammatory factors IL-6, IL-12, and TNF-α in BMDCs.

## 4. Discussion

Parasite-derived exosomes play an important role in the exchange of information among parasites and in the interaction between parasites and their hosts. Parasite miRNAs and antigens carried by parasite-derived exosomes play a vital role in these processes. DCs can potently activate naive T cells, initiate primary immune responses, and determine the direction of the body’s immune response during a parasitic infection. *E. granulosus* ES antigens regulate the maturation and function of DCs, affect the release of inflammatory factors, and regulate Th1/Th2 immune responses [7]. However, the functions and mechanisms of both *E. granulosus* PSC-ELVs and their abundant miRNAs toward DCs have not yet been reported.

We isolated and identified PSC-ELVs and found that *E. granulosus* miRNAs could be transported into BMDCs to regulate immune responses via the uptake of PSC-ELVs by BMDCs. Many studies have demonstrated that ELVs can be internalized by DCs, transferring their contents, e.g., miRNAs, into the DCs cytosol to regulate immune responses [26,27,28]. Parasite infection initially promotes the production of stimulatory DCs to help the host to kill parasites, while it will gradually induce the production of regulatory DCs to help the parasites to evade immune responses. Immature DCs differentiate into stimulatory DCs when stimulated by proinflammatory signals such as pathogen-associated molecular patterns and secrete proinflammatory factors such as IL-6, IL-12, IFN-γ, and TNF-α. However, under the stimulation of inhibitory signals such as TGF-β and IL-10, they differentiate into regulatory DCs and secrete anti-inflammatory molecules such as TGF-β, IL-10, ARG, and IDO [14].

To the best of our knowledge, this is the first study to show that PSC-ELVs can induce immature DCs to differentiate into stimulatory DCs. *E. granulosus* PSC-ELVs were found to induce BMDCs to produce high levels of IL-6, IL-12, TNF-α, IL-1β, and IFN-γ and low levels of IL-10, which are clear proinflammatory characteristics. In addition, co-stimulation with PSC-ELVs and LPS had a synergistic effect, inducing BMDCs to produce higher levels of these cytokines. Furthermore, PSC-ELVs induced BMDCs to upregulate the expressions of MHCII, CD40, CD80, and CD86 molecules on their surface, which helped to promote BMDCs maturation. The percentage of DCs was found to increase in the liver leukocytes of mice infected with *E. granulosus*. Moreover, a previous related study demonstrated that these DCs highly express ARG-1, which inhibited the T cell response to *E. granulosus* antigens [4]. Therefore, *E. granulosus* PSC-ELVs induce the production of stimulatory DCs, which might serve as a new strategy to enhance the anti-parasitic immunity. However, the immune functions of *E. granulosus* PSC-ELVs and PSC-derived ES are likely different. *E. granulosus* adult worm antigen (AWA) and adult ES, both derived from the adult stages of *E. granulosus*, trigger different immune responses [7]. Research has shown that PSC-derived ES can significantly inhibit proinflammatory responses by directly inducing B10 cells and inhibiting B17 and Th17 cells, thereby downregulating anti-parasitic immunity [29,30]. Therefore, in the current study, it was speculated that PSC-ELVs may have different immune effects and might carry *E. granulosus*-related virulence factors that promote the maturation and differentiation of DCs, thus having the potential to induce Th1-type immune responses and even enhance host immunity.

NF-κB1 is necessary for DCs to promote optimal Th2-type responses, which might have therapeutic potential for helminthiasis dominated by Th2 immune responses [31]. Furthermore, our study found that egr-miR-277a-3p in PSC-ELVs might induce the polarization of BMDCs toward stimulatory DCs by directly targeting *Nfkb1*. egr-miR-277a-3p significantly upregulated the mRNA levels of proinflammatory factors *IL-6*, *IL-12,* and *TNF-α* and downregulated the anti-inflammatory factors *IL-10* and *ARG-1* in BMDCs, which might help BMDCs differentiate into stimulatory DCs. *Nfkb1* was found to be a potential direct target gene of egr-miR-277a-3p using qRT-PCR, western blotting, and Luciferase reporter assays. Meanwhile, in the current study, it was found that egr-miR-277a-3p could significantly upregulate the expression of NF-κB p65 in BMDCs. NF-κB can mediate inflammatory responses, and an increase in p65/p50 expression promotes the secretion of IL-6 and TNF-α [24,32]. Therefore, it was speculated that egr-miR-277a-3p could directly target *Nfkb1* and might induce the production of these proinflammatory cytokines by modification of the NF-kB p65/p50 ratio in BMDCs. As such, the blockade of NF-κB1 might prove valuable in DC-based therapies for echinococcosis.

This study did have some limitations, as it did not explore whether egr-miR-277a-3p could promote the maturation and differentiation of DCs in vivo. Therefore, the injection of an egr-miR-277a-3p agomir into *E. granulosus*-infected mice or NF-κB1 deficient mice to confirm the functions of egr-miR-277a-3p will be included in a future study.

## 5. Conclusions

In conclusion, this study found that PSC-ELVs could promote BMDCs maturation and differentiation into stimulatory DCs, which is beneficial to mediating a Th1 immune response. Further, egr-miR-277a-3p could directly target *Nfkb1* to suppress its expression, which induced DCs to produce proinflammatory cytokines in a cross-species manner. Therefore, PSC-ELVs and egr-miR-277a-3p could regulate the host immune response, thus providing a new strategy to prevent and treat echinococcosis.

## Data Availability

The original data supporting the conclusions of this study are included in the manuscript and Supplementary Materials.

## Supplementary Materials

The following supporting information can be downloaded at https://www.mdpi.com/article/10.3390/cells11203220/s1, Figure S1: Standard curve of the BSA standard solution; Table S1: Primers used for qPCR detection of miRNAs; Table S2: Primers used for qPCR detection of mRNAs.
