# Supplementary material for: Echinococcus granulosus Protoscoleces-Derived Exosome-like Vesicles and Egr-miR-277a-3p Promote Dendritic Cell Maturation and Differentiation
Source: Cells. 2022 Oct 14;11(20):3220. doi: 10.3390/cells11203220 (PMC9600664; doi:10.3390/cells11203220)
Supplement: Supplementary file 1 [file cells-11-03220-s001.zip › Supplementary Table S2 Primers used for qPCR detection of mRNA.pdf]

Supplementary Table S2 Primers used for qPCR detection of mRNA

Gene	Forward Primer (5'-3')	Reverse Primer (3'-5')
GADPH	GAGCCAAACGGGTCATCATCT	GAGGGGCCATCCACAGTCTT
IL-6	ACAACCACGGCCTTCCCTACTT	CACGATTTCCCAGAGAACATGTG
IL-10	CCAAGCCTTATCGGAAATGA	TTCACAGGGGAGAAATCG
IL-12	GGAAGCACGGCAGCAGAATA	AACTTGAGGGAGAAGTAGGAATGG
IL-1β	TCACAGCAGCACATCAACAA	TGTCCTCATCCTGGAAGGT
TNF-α	AAGCCTGTAGCCCACGTCGTA	GGCACCACTAGTTGGTTGTCTTTG
IFN-γ	AAAGAGATAATCTGGCTCTGC	GCTCTGAGACAATGAACGCT
iNOS	CCAAGCCCTCACCTACTTCC	CTCTGAGGGCTGACACAAGG
Arg-1	CTCCAAGCCAAAGTCCTTAGAG	AGGAGCTGTCATTAGGGACATC
TGF-β	GACCGCAACAACGCCATCTA	GGCGTATCAGTGGGGGTCAG
IDO	AGCAATCCCCACTGTATCCA	GGTCCACAAGTCACGCATC
PD-1	GCCTGGCTCACAGTGTCAG	TCCAGGGCTCTCCTCGATT
NF-κB p65	ATGGCTACTATGAGGCTGACCTC	TGCCGATGCACATCAGCTTGAG
NF-κB1	CAACCAAAACAGAGGGGATT	TTGTGACCAACTGAACGATA
