# Supplementary figures and images for: Echinococcus granulosus Protoscoleces-Derived Exosome-like Vesicles and Egr-miR-277a-3p Promote Dendritic Cell Maturation and Differentiation
Source: Cells. 2022 Oct 14;11(20):3220. doi: 10.3390/cells11203220 (PMC9600664; doi:10.3390/cells11203220)

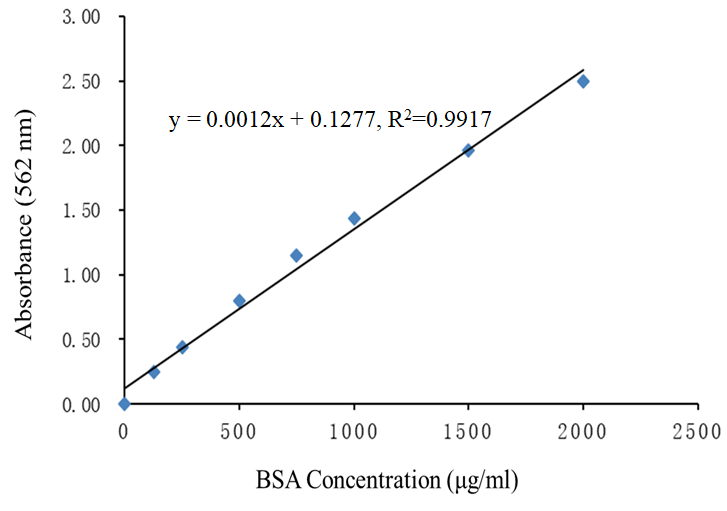

Supplement: Supplementary file 1 [file cells-11-03220-s001.zip › Supplementary Figure S1 Standard curve of BSA standard solution.jpg]
